# Triclosan and Its Consequences on the Reproductive, Cardiovascular and Thyroid Levels

**DOI:** 10.3390/ijms231911427

**Published:** 2022-09-28

**Authors:** Ana C. Marques, Melissa Mariana, Elisa Cairrao

**Affiliations:** 1 Health Sciences Research Centre (CICS-UBI), University of Beira Interior, 6200-506 Covilhã, Portugal; 2 Faculty of Health Sciences (FCS-UBI), University of Beira Interior, 6200-506 Covilhã, Portugal

**Keywords:** triclosan, antimicrobial, endocrine disruptor, reproductive system, cardiovascular system, thyroid

## Abstract

Hygiene is essential to avoid diseases, and this is thanks to daily cleaning and disinfection habits. Currently, there are numerous commercial products containing antimicrobial agents, and although they are efficient in disinfecting, it is still not known the effect of the constant use of these products on human health. In fact, a massive use of disinfectants has been observed due to COVID-19, but the possible adverse effects are not yet known. Triclosan is one of the antimicrobial agents used in cosmetic products, toothpaste, and disinfectants. This compound is an endocrine disruptor, which means it can interfere with hormonal function, with its estrogenic and androgenic activity having already been stated. Even if the use of triclosan is well-regulated, with the maximum allowed concentration in the European Union of 0.3% (*m*/*m*), its effects on human health are still uncertain. Studies in animals and humans suggest the possibility of harmful health outcomes, particularly for the reproductive system, and in a less extent for the cardiovascular and thyroid functions. Thus, the purpose of this review was to analyse the possible implications of the massive use of triclosan, mainly on the reproductive and cardiovascular systems and on the thyroid function, both in animals and humans.

## 1. Introduction

Triclosan (TCS), 5-chloro-2-(2,4-dichloro phenoxy) phenol, is an endocrine-disrupting chemical often used as an antiseptic, disinfectant, or preservative [1,2,3,4,5,6].

An endocrine disruptor is a chemical substance that causes adverse effects in an organism by disrupting its endocrine function [7]. Many are chemicals present in the environment, which can be natural or synthetic and that can alter the endocrine functions by mimicking or inhibiting the action of endogenous hormones [8]. Exposure to these compounds can lead to alterations in one or more signalling pathways, that may induce negative effects on reproduction, growth, survival, behavioural, and obesity. These changes can be passed on to subsequent generations [9,10].

In the European Union, 85% of triclosan is used in personal care products (e.g., soaps, toothpaste, mouthwash, and cosmetics), and 10% in plastics and food contact materials (household utensils), while the remaining 5% is used in textiles (antibacterial clothing) [1,2,3,4,5,11]. It is also used as a suture coating, but only contributes 0.00016% of the overall TCS global exposure [12]. It has been used for these purposes since its introduction in 1968 [1]. Despite being used as a constituent of several products, there are still no defined regulations for TCS as it is considered tolerable and safe. Most personal care products contain between 0.1–0.3% (*w*/*w*) of triclosan [13]. As for its regulation, the European Commission and the Infarmed impose a maximum of 0.3% [6,14], while the FDA (U.S. Food and Drug Administration) has been decreasing the maximum allowed over the years [15].

Some studies presented by the European Chemicals Agency have concluded that triclosan can interfere with estrogenic (ER) and androgenic (AR) receptors and that it has adverse effects on the thyroid and cardiovascular systems. Specifically, these studies have led to the discovery of the vasorelaxant properties of triclosan, of the effects that this compound may have in miscarriages or malformations of a foetus [16] and its potential to interfere with the thyroid hormones due to their structural similarity with TCS [17]. Besides the ERs and Ars, there are also other receptors that can be influenced by TCS. The aryl hydrocarbon receptor (AhR), a transcription factor that activates gene expression in a ligand-dependent manner, can modulate the function of oestrogen and androgen receptors, which means they are in crosstalk. TCS was previously tested with AhR because of its structural similarity to AhR-ligands, where it was discovered that TCS is a partial agonist of AhRs. Thus, it can be assumed that TCS agonistic activity toward AhR can influence ER and AR because of the crosstalk between these three receptors. A different receptor also affected by TCS is peroxisome proliferator-activated receptor gamma (PPARγ). This receptor is expressed in several reproductive tissues, as well as in rodent and human placentas, being involved in different metabolic processes. It was previously shown that PPARγ and AhR are in crosstalk since PPARγ agonists affect the expression of AhR and that AhR agonists affect PPARγ expression. Considering that TCS is a partial agonist of AhR and a weak agonist of PPARγ, it will affect the expression of both AhR and PPARγ. Thus, knowing that TCS interacts with all of these receptors, it is expected that TCS can influence the crosstalk between PPARγ, AhR, ERs and ARs [18,19,20,21,22].

Currently, the literature is still unclear about the potential effects of TCS on organisms and humans, and the impact on human health of the passive use of these compounds, mainly during the COVID-19 season, is still completely unknown. Therefore, and based on this assumption, the aim of this review was to analyse in more detail the possible implications of the massive use of triclosan, mainly on the reproductive and cardiovascular systems and on the thyroid function in both animals and humans.

## 2. Physical and Chemical Properties of Triclosan

Triclosan is a white, odourless, tasteless, crystalline powder with a molecular weight of 289.5 g/mol. It is commercially known as Irgasan DP 300 and Irgacare MP for oral applications [23,24].

Structurally, TCS is a molecule with functional groups for phenol ether and it has a lipophilic nature (log K_ow_ = 4.8), which suggests that it bioaccumulates in adipose tissue [25]. The chemical structure is a halogenated biphenyl ether which confers similar properties to many other toxicants, for example, polychlorinated biphenyls (PCBs), polybrominated diphenyl ethers (PBDEs), bisphenol A (BPA) and dioxins. It is a chlorinated phenoxy-phenol compound with a pKa of 8.1. It does not hydrolyse easily; however, the pH, the presence of metals and dissolved organic matter can affect the photosensitivity of triclosan [24] (Table 1).

It is almost insoluble in water, relatively soluble in alkaline solutions and soluble in most non-polar organic solvents (Table 2).

Moreover, TCS also has impurities associated with it such as copper, mercury, lead, and cadmium, among others [6].

It is a broad-spectrum antimicrobial agent whose activity depends on its concentration and formulation, that is, at low concentrations, TCS is bacteriostatic while at high concentrations it is bactericidal. This antibacterial effect has been widely studied and was demonstrated to be related with the inhibition of a specific enzyme in fatty acids biosynthesis, the enoyl-acyl carrier protein reductase (ENR). This inhibition will suppress the membrane phospholipids and the proliferation of microbial cells. TCS has been considered safe for humans and animals since ENR is absent in eukaryotes; however recent studies demonstrate that TCS is considered toxic for eukaryotes [30,31]. It acts primarily on the cytoplasmic membrane and its entry into the cells appears to be by diffusion [6,23,32,33] due to the lipophilic properties of TCS. These allow TCS to be incorporated into the phospholipid bilayer, which will disturb phospholipid packing resulting in the formation of lipid pores and alterations in the membrane permeability to ions and larger molecules. This mechanism is known as the TCS membranotropic effect. In 2003, Lygre et al. demonstrated that this was the mode of action by which TCS exerts its toxic effect on eukaryotes, destabilizing the membrane of a cell or the mitochondria [30,31,34].

This compound has a half-life of approximately 11 days at the water surface and is degraded in aerobic soil with a half-life of 18 days [2].

## 3. Metabolism

TCS can be absorbed through several routes, including the mucous membrane of the oral cavity, dermal exposure, or the gastrointestinal tract. Considering that most products containing TCS are used for dermal application, its main route of absorption is the skin, through which it is rapidly absorbed due to its lipophilic properties [35] (Table 3). Additionally, Wu et al. noted that TCS can also be absorbed orally which leads to the detection of metabolites in plasma, and also through the gastrointestinal tract [32].

Concentrations in the millimolar range have been detected in humans; therefore, considering the amount of TCS found in human consumer products, all exposure routes are important for the final exposure [38]. In a study presented in 1992 [39] when triclosan (1.6 mg) was applied directly to the skin of rats, it was rapidly absorbed and the concentration peak occurred between 12 and 18 h following exposure. Further to this study, Moss, Howes and Williams demonstrated that triclosan penetrates faster through rat skin than through human skin and that after 24 h only about 12% of the dose was on human skin and about 26% on rat skin [40].

Regarding the distribution of this substance, the studies currently available only concern rodents. Kanetoshi et al. were the first to demonstrate the metabolization of this substance in the bile and the liver. The authors demonstrated that within 24 h of TCS administration, the highest levels were present in the bile (33.6 µg TCS/g tissue), followed by the liver which contained about 3.0 µg TCS/g tissue. Concentrations in these two tissues remained high for 24 h after administration when compared to other tissues [39].

In another study performed using rats, this compound was detected in the 15 tissues analysed 12 h after a skin application, with the gallbladder and bladder containing the highest concentrations while the testes, thymus and brain had the lowest concentrations [41]. Similarly, Geens et al. extracted adipose tissue, brain tissue and liver from eight men and three women (aged between 9 and 64 years) by autopsy, and measured TCS. These authors concluded that the liver and adipose tissue were better retainers of TCS than brain tissue [42].

Regarding metabolism itself, TCS can be metabolised by three pathways: cytochrome P450 (P450), UDP-glucoronyltransferase (UGT) and sulphotransferase (SULT). The latter two are part of the phase II metabolism of xenobiotics [26,43].

**Figure 1:**
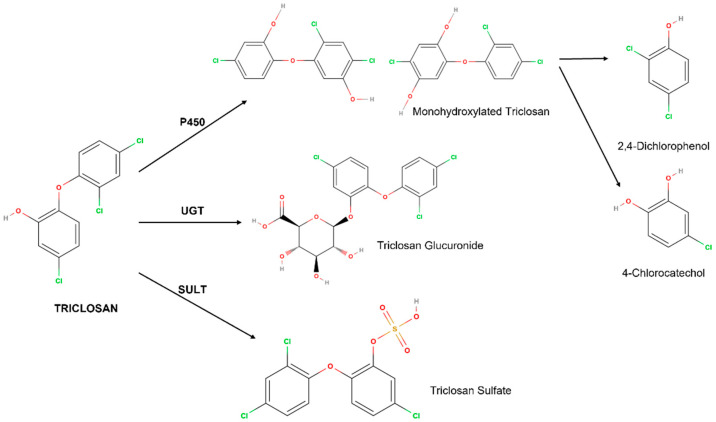
Metabolism of triclosan and its metabolites.

The main metabolite of TCS hydroxylation is triclosan monohydroxylate. This in turn can be cleaved to 2,4-dichlorophenol and 4-chlorocatechol. A fourth metabolite can also be formed from the hydroxylation of 2,4-dichlorophenol, forming 3,5-dichlorocatechol [44] (Figure 1).

The fact that there are two pathways of the phase II metabolism is not influenced by the route of administration; however, the ratio between the transformation of TCS by the two pathways is already influenced by the species [25,45].

Glucuronidation and the sulphation of TCS causes the addition of glucuronic acid and sulphate, respectively, to the hydroxyl group, thus destroying the proton translocating nature and adding a charged/polar group to the TCS. This in turn will enhance its hydrophilic properties and decrease TCS’s ability to accumulate in adipose tissue [3].

The liver is the main site of TCS metabolism; however, it also occurs at minimal levels in the skin. It is primarily metabolized through the SULT pathway, yet 24 h after administration, both phase II metabolites are found. Despite undergoing metabolization, the unmetabolized form of TCS remains the predominant form [40].

In the liver, the production of metabolites depends on the dose of TCS. For exposures of 1–5 µM of TCS for 30 min, there was an equal production of TCS-sulphate and TCS-glucuronyl, and for exposures greater than 20 µM the glucuronidation predominated while for doses below 1 µM it led to sulphation [46].

In a 2014 study, 46 volunteers, namely, 26 men and 20 women aged between 4 and 80 years, provided urine samples which were tested and both TCS-sulphate and TCS-glucuronyl were detected. The main metabolite identified was TCS-glucuronyl [47].

Regarding the elimination of TCS, the main route of excretion is through urine (57–87% of an administered dose) and faeces is the secondary route (5–33% of an administered dose). This compound is excreted mainly in its metabolised forms: TCS-glucuronic and TCS-sulphated; thus, during the first 4 days after an exposure, between 24% and 83% of the TCS consumed is excreted, and only after 8 days does it reach basal excretion levels [3,45,48].

Triclosan has already been detected in several matrices, such as in urine, blood, serum, and breast milk (Table 4). For this reason, some studies have been conducted to determine whether or not the presence of TCS can affect the foetus, through crossing the placental barrier, or even if it can induce adverse effects in future generations, as already observed for other endocrine disruptors [49,50,51].

## 4. Effects on the Reproductive System

### 4.1. Animals

TCS has been widely studied in animals at the level of the reproductive system. It is also known that in this system, the effects are mainly due to the modulation of androgenic and estrogenic receptors.

In a study conducted by Ishibashi et al., several concentrations of triclosan were tested on embryos and adults of the *Oryzias latipes* fish. At concentrations of 625, 1250 and 2500 µg/L, no embryos were born, and they found that at 313 µg/L the incubation time increased significantly; however, fertility was not affected during the study [57].

Liu et al. used zebrafish (*Danio rerio*) to test the toxic effects of three concentrations of TCS (0.08, 0.16 and 0.25 mg/L) on the liver. The growth of the zebrafish showed no significant changes; however, there was an increase of 11.67–19.16% in the zebrafish weights and of 21.53–47.42% in the liver weights at exposures of 0.16 and 0.25 mg/L. At these concentrations, the superoxide dismutase (SOD) activity decreased while the malondialdehyde (MAD) levels gradually increased with different TCS concentrations. Thus, the authors demonstrated that exposure to TCS causes oxidative stress in the liver depending on the exposure time and concentration. This exposure created several hepatocellular changes, an increased hepatic plaque gap, necrosis, and atrophy, with the last two being concentration dependent. The same authors also observed a TCS concentration-dependent apoptosis and they quantified the expression of Bcl-2 and Bax to better understand which pathway of apoptosis was promoted by TCS. The expression of Bcl-2 at concentrations of 0.16 and 0.25 mg/L was significantly decreased while Bax was increased at concentrations of 0.08 and 0.25 mg/L. This relationship between Bax and Bcl-2 led the authors to hypothesise that hepatocyte apoptosis is directly related to the exposure of zebrafish to TCS [58].

Recently, Qiao et al. also tested 2, 20 and 200 µg/L of TCS in zebrafish, observing no mortality or any abnormality in their behaviour during the experiment. Fertilisation was not affected at any of the concentrations; however, the births decreased. Furthermore, in males, the testosterone levels decreased with the higher TCS concentration while the oestradiol and vitellogenin (VTG) levels increased in the 20 and 200 µg/L of TCS groups. On the other hand, in females, there was only a decrease in the VTG levels at 20 and 200 µg/L of TCS. These results indicate that TCS has an estrogenic influence in zebrafish, with males being more sensitive than females. There was also a significant decrease in mature spermatozoa and destruction of the testis structure at the concentration of 200 µg/L of TCS, and in females, an increase in the number of immature oocytes was observed at the same concentration [59].

Another model widely used for in vivo studies are rats. Kumar et al. observed that the administration of 5 mg/kg/day to male Wistar rats did not induce a significant change in the testes weight, but higher doses of TCS induced a significant change in the testes and sexual tissues. The weight of the testes, epididymis, ventral prostate, vas deferens and seminal vesicles decreased by 20–50% with 10 mg/kg/day and 35–49% with 20 mg/kg/day. The authors also quantified several hormones and observed a decrease of 38.5% in the luteinizing hormone (LH), 17% in the follicle stimulating hormone (FSH), 35% in cholesterol, 31% in pregnenolone and 41% in testosterone after a dose of 20 mg/kg/day [60].

Regarding the study of gender differences in reproduction, in the following year, 2010, Stoker et al. performed a daily administration of TCS (9.375; 37.5; 75; 150 mg/kg) to female Wistar rats, between the Postnatal day (PND) 22 and the PND 42 (PND = 0, day of birth of the females used in the study), to examine the effects of TCS on female pubertal development. They analysed if the time until the opening of the vaginal canal was affected, as well as the histology of the uterus and ovaries, and some hormones including E2 and LH. The authors observed that this compound altered the reproductive development and response to exogenous oestrogens. They also observed a lower age for the opening of the vaginal canal with the administration of 150 mg/kg, which was an estrogenic response of the triclosan since stimulation through oestrogens is required for the canal to open. The serum oestradiol concentrations at PND 42 were decreased followed by administration of TCS at 37.5 mg/kg and 150 mg/kg, while the LH levels did not show significant changes. Thus, the study suggested that TCS increases oestrogen activity with a potential to alter the oestrogen-dependent functions. In summary, the authors concluded that TCS affects the reproductive development and uterine responses to exogenous oestrogens in developing females [61].

Two years later, in 2012, Jung et al. analysed the estrogenic activity of TCS using well-established models, both in vivo and in vitro. For the in vivo approach, female Sprague-Dawley rats were treated with 17α-ethinyl-oestradiol (1 mg/kg) and TCS (7.5, 37.5 and 187.5 mg/kg), between PND 19 and 21. The TCS increased the uterine weight and mRNA expression for complement 3, which is an oestrogen-sensitive gene in the uterus, in immature mice after a treatment with TCS at 37.5 mg/kg and 187.5 mg/kg. This shows that TCS has estrogenic activity. In vitro, the estrogenic activity upon TCS exposure was analysed on the expression of calbindin-D9k (CaBP-9k), which is a biomarker for the detection of endocrine disruptors in GH3 cells (i.e., Wistar rat epithelial cells). The expression of CaBP-9k was increased in the presence of TCS, which demonstrates the endocrine disrupting nature of TCS [62]. In a similar study by Louis et al., also using female Wistar rats between PND 19 and 21, doses of ethinyl-oestradiol (0.125, 0.25, 0.5, 1, 2 and 3 µg/kg) or ethinyl-oestradiol combined with TCS (2.3, 4.69, 9.375 and 18.75 mg/kg) were administered orally. No changes in the body weight were observed; however the uterine weight was increased when ethinyl-oestradiol (1, 2 and 3 µg/kg) was combined with TCS (4.69, 9.375 and 18.75 mg/kg). The expression of CaBP-9K was also analysed, but they found no changes when treated with TCS alone; however, the coupling of ethinyl-oestradiol and TCS caused an increase in its expression, but not significantly higher than the expression increase with the ethinyl-oestradiol alone. The authors concluded that TCS can alter the uterine response in the presence of low concentrations of ethinyl-oestradiol [63].

Regarding the effects of TCS in *Mus musculus* rats’ gestation, Crawford et al. compared several doses of TCS (0, 87, 262, 523 and 785 mg/kg) with BPA. Females receiving the TCS dose on gestational days 0 and 1 showed no significant differences in the implantation sites when compared with the control. On gestational day 2, significant differences were already observed for the 785 mg/kg triclosan dose and on day 3 for the 523 mg/kg triclosan dose. When exposed to 18 and 785 mg/kg of triclosan from gestational days 1–3, the number of implantation sites decreased significantly on gestational day 6. The number of implantation sites was also reduced after an injection of these two doses on gestational day 3 and only of the higher dose on gestational day 2 [64]. Thus, this study concluded that TCS may affect intrauterine implantation when administered in combination with BPA.

In a different study, 3-month-old rats were administered with 1, 10 and 100 mg TCS/kg/day from gestational day 6. The TCS levels were measured up to gestational days 11 and 16. An exposure to TCS demonstrated a dose-dependency in increasing foetal death. The incidence of spontaneous abortion at doses 10 and 100 reached 60% and 80%, respectively. The level of oestrogen sulphotransferase protein (EST) was decreased at gestational day 16 at the highest dose, as were its plasma and placental activities. These abortions were most likely caused by the inhibition of EST activity leading to placental thrombosis and degeneration [65]. Thus, the authors concluded that higher levels of TCS may lead to the occurrence of spontaneous abortions due to the inhibition of EST activity.

One year later, in 2016, Feng et al. recorded the total and uterine weights of female mice throughout gestation after an administration of TCS (0, 30, 100 and 300 mg/kg), and noted a lower weight gain in the groups where 300 and 600 mg/kg of TCS was administered when compared to the control; however, significant differences were only found regarding the uterine weight in the 600 mg/kg group. The tissues presenting the highest concentration of TCS were the placenta, liver, and kidneys with 12.83, 9.52 and 8.74 µg/g, respectively. In the placenta, the mean TCS levels following 0, 30, 100 and 300 mg/kg of TCS administrations were 0.033, 13.05, 28.23 and 55.83 µg/g, respectively. The LH and FSH levels were identical to the control group, whereas the levels of human chorionic gonadotropin (hCG), prolactin, progesterone, oestradiol, and testosterone were significantly reduced in all the exposure groups when compared to the control. Thus, the placenta may be a target of TCS in rats during the gestation period [66].

In the same way, a study performed by Montagnini et al. tested the effect of low doses of TCS (0.8, 2.4, and 8.0 mg/kg) over the course of three generations of Wistar rats named F0, F1 (the offspring of F0) and F2 (the offspring of F1). The TCS was orally administered to the F0 generation only between PND 49 and 120 in females, while in males it continued until PND 140. Thus, weight changes, food consumption during the pre-mating period, sexual behaviour assessment, male organs of the F1 generation, plasma testosterone quantification, all sperm parameters, testicular histomorphometry, sexual and physical development of the F1 and F2 generations and neuronal behavioural tests were accounted for. They concluded that these concentrations of TCS had no significant effect other than a reduction in the viability and motility of spermatozoa from the F1 generation being administered with 2.4 mg/Kg at PND 140. Doses administered between PND 49 and PND 140 did not affect the sperm parameters; therefore, the authors concluded that TCS could have an impact on gametogenesis during the foetal period [67].

More recently, Raj et al. analysed the effects of accumulated TCS on the histopathology and secretory functions of the epididymis, seminal vesicle, and sperm indices in adult Swiss-strain rats. Four different doses of triclosan (40, 80, 160 and 320 mg/kg) were administered for 42 consecutive days. All the concentrations significantly decreased the epididymal and seminal vesicle weights and led to a decrease in the sperm percentage and viability, and sperm count; furthermore, the percentage of non-viable spermatozoa increased. The histology of the epididymis was marked with changes throughout its length with disorganization and the appearance of vacuoles. The final chromatographic study showed an accumulation of TCS in the epididymis and seminal vesicle. Thus, Raj et al. concluded that TCS caused alterations in the epididymis and seminal vesicle, as well as in the sperm indices, epididymal sialic acid levels and fructose levels of the seminal vesicle [68].

Concerning the effect of this compound on the placenta, in 2010, TCS was tested on the placental tissue from ewes with gestation periods ranging between 126–130 days. In this study it was demonstrated that the TCS was a potent inhibitor of placental EST activity. This inhibition was analysed, and the authors observed that it occurred mainly competitively; therefore, it can be assumed that TCS occupies the substrate binding sites. The role of placental EST is not yet fully known; however, alterations in its expression are thought to be associated with spontaneous abortions and foetal loss [69], as previously demonstrated in rats by Wang et al. [65].

In summary, TCS has a variety of reproductive effects in fish, rats and even sheep. The decreased fertility beyond the induction of abortion was the most observed consequence in any of the models. This may be due to the way the substance acts on the receptors or on oestradiol concentrations, as observed by Wang et al., where they demonstrated an inhibition of EST activity which caused a thrombosis in the placenta. Furthermore, TCS also appears to affect the serum concentration of several hormones such as LH, FSH, hCG, pregnenolone and cholesterol, which can lead to reproductive problems such as foetal loss and decreased fertility in subsequent generations.

### 4.2. Humans

In humans, TCS has not been widely studied yet, with most of the existing studies being related to the hormonal levels of EST, FSH and the effects at the foetal-placental level, namely, spontaneous abortions.

The first authors to demonstrate that TCS could be associated with adverse reproductive effects were Wang et al., finding an association between urinary TCS concentration levels and increased spontaneous abortions. These authors recruited a population of 452 women at 14 and 24 weeks of pregnancy with mean ages of 28 and 27 years, respectively. Their E2 levels at low-TCS concentrations were lower than the control; however, at high-TCS levels there were no changes. The plasma EST levels were reduced at both low- and high-TCS concentrations, while the EST activity at high-TCS concentrations was lower compared to the control and the low-TCS concentrations [70]. This increase in the number of abortions at higher TCS concentrations may be due to decreased EST activity caused by the TCS, as it was already demonstrated in animals.

Miscarriages may be linked to an inhibition of the placental 11β-HSD2 enzyme. In this sense, Zhang et al. observed that triclosan altered the expression of 11β-HSD2 in human syncytiotrophoblasts and examined the apoptotic mechanism induced by TCS. At concentrations of 0.1 µM and higher of TCS, the mRNA levels decreased significantly, and at the protein level there was a significant inhibition over a similar range of concentrations. There was also a decrease in the viability of syncytiotrophoblasts in a concentration dependent manner (0.001–10 µM), with statistical significance at concentrations above 0.1 µM. Moreover, the authors observed that triclosan induced the apoptosis of syncytiotrophoblasts and that the inhibition of 11β-HSD2 was explained by the induction of apoptosis [71].

In 2017, an epidemiological study also demonstrated the association between the urinary TCS levels of 401 pregnant women (at 16 weeks of gestation) and birth weight, length, head circumference and length of gestation. Urine was collected at 16 and 27 weeks of gestation, and TCS was detected in 91% and 83% of the samples, respectively, with a mean concentration of 16 ng/mL. This study showed a positive association between the urine triclosan concentration and a moderate reduction in birth weight, length, head circumference and gestational length [72].

Two years later, Jurewicz et al. analysed the association between triclosan concentration and ovarian reserve, which is a marker of female fertility. They counted the antral follicles and quantified the anti-müllerian hormone, follicle stimulating hormone and oestradiol. The authors recruited 511 menstruating women (25–39 years old) that provided blood and urine samples. The blood samples were taken at the beginning of the menstrual cycle, approximately between the second and fourth day, so that the hormones could be counted, while the antral follicle counts were performed in both ovaries during the beginning of the follicular phase of the menstrual cycle. Urine TCS concentrations (0.3–1677.68 ng/mL) were shown to be negatively associated with the antral follicle count, while the anti-müllerian hormone, follicle stimulating hormone and oestradiol did not show a statistic relevance. Thus, the authors concluded that TCS may be associated with decreased female fertility, although further studies are needed to demonstrate the mechanism involved in this effect [73].

The following year, Yuan et al. compared the levels of TCS present in urine with the sperm quality in a Chinese population, between 2018 and 2019. From the 406 subjects (aged between 21 and 56), 57.1% had never smoked and 83.5% had never consumed alcoholic beverages. The parameters analysed included the sperm volume, concentration, count, total, progressive and non-progressive motility, immobility, percentage of hyperactivated sperm, mean velocity, curvilinear velocity, straight line velocity, lateral head deviation amplitude, linearity, sway, beat cross frequency and straightness. TCS was detected in 74.6% of the samples with a mean of 1.7 µg/L. None of the parameters studied were associated with TCS exposure, although a higher TCS exposure showed higher motility parameters indicating a potential non-linear influence of TCS on sperm quality [74].

In summary, the effects caused by TCS in humans are very similar to those observed in animal models. Spontaneous abortion caused by chronic exposure to TCS is the most observed consequence, although the specific pathway by which it occurs is not yet known. Furthermore, sperm motility is also disrupted by TCS exposure. Thus, we can conclude that fecundity/fertility ends up being the most affected by exposure to TCS due to its influence on both sperm and eggs, which may be a cause of the increased infertility present in developed countries.

## 5. Effects on the Cardiovascular System

### 5.1. Animals

Regarding the cardiovascular system, the effect of TCS has not yet been studied as much as the reproductive system; however, some effects on the heart rate and cardiovascular morphology are already known.

In a study conducted by Saley et al., zebrafish were exposed to 1mL of 0, 0.4, 40 or 400 µg/L doses, between 8 and 120 h post fertilisation (hpf), and the dose was changed every 24 h. Doses below 40 µg/L showed minimal changes while a 400 µg/L dose resulted in a dilated auricle, smaller ventricles and unlooping of the heart. The same concentrations were used to assess the cardiac function and structure. The heart rate was reduced by 30% and the beat volume by 40%, which resulted in an output decrease of 60% at the highest dose. At the highest dose, the diastolic end volume was reduced by 17% whilst the systolic volume was unaffected, with 80% of the embryos showing regurgitation [75]. Thus, the authors showed that TCS induces morphological changes in the heart and changes in heart rhythm, namely, the heart rate.

Once more in zebrafish, Wang et al. studied the influence of TCS and its derivatives, 2,4,6-trichlorocatechol and 2,4-dichlorophenol, in cardiotoxicity. The authors used the three compounds in a mixture named TCS-DT at 0.28, 0.56 and 0.84 mg/L, during 6 to 120 hpf in larvae, and they also used 0.14, 0.28 and 0.56 mg/L between 30 and 90 days after fertilisation in adult zebrafish. At 48 hpf, the heart rates of the larvae were significantly reduced in the 0.56 and 0.84 mg/L treatments and the ejection fraction showed a concentration-dependent decrease. At 120 hpf, the looping distance from the atrium to the ventricle increased significantly with the concentration; thus, the TCS-DT exposure affected the cardiac looping and led to abnormal ventricles influencing the cardiac function. Moreover, the creatinine kinase MB and triglyceride levels in the adult hearts increased at all concentrations, the SOD levels increased at 0.14 and 0.28 mg/L, but decreased at 0.56 mg/L, while the lactate dehydrogenase (LDH) levels increased at 0.14 mg/L and decreased with the remaining concentrations. The authors concluded that changes in these activities led to damage in the cardiomyocytes, suggestive of myocarditis. Overall, this demonstrates that exposure to TCS-DT affects normal cardiovascular growth and development in zebrafish [76].

Ma et al. studied the relationship between a TCS-induced abnormal expression of miR-181a-5p and vascular development. The study began by analysing which miRNAs expressions were affected by TCS exposure, which was miR-181a-5p. Zebrafish embryos were treated with 62.5, 125 and 250 µg/L of TCS between 4 and 120 hpf. At the lowest concentration, there was only a small impact on the vascular development and distribution; however, at the other concentrations, a gradual decrease in the blood flow was noted. For 250 µg/L of TCS, the blood flow was mostly confined in the head; however, there was no distribution of the blood vessels or blood flow in the forebrain and diencephalon. The distribution of red blood cells decreased by 60% in the group treated with 250 µg/L. Additionally, exposure to TCS led to an increase in the miR-181a-5p expression. When miR-181a-5p inhibitors were injected, the number of blood cells increased, while an injection of an analogue of miR-181a-5p decreased the number of blood cells. Thus, the authors concluded that the increased expression of miR-181a-5p was due to exposure to TCS, causing atrophy of the blood vessel development in zebrafish [77].

Cherednichenko et al. analysed the effect of TCS on ryanodine 1 and 2 receptors in rats. After an administration of 6.25, 12.5, or 25 mg/kg of TCS, the authors observed several cardiovascular deficits in the rats including a reduced cardiac output, reduced diastolic volume in the left ventricle, and a reduced maximum time derived from the left ventricular pressure development. The highest dose group exhibited a 25.3 ± 15.7% decrease of the cardiac output, implying that TCS had severe cardiovascular effects. There was an 18% decrease of the exerted force which was significantly lower than the vehicle and placebo groups after a TCS dose of 40 mg/kg. These deficits were reversible as the rats regained full strength 24 h after the TCS administration [78].

More recently, the effect of TCS on vascular contractility was evaluated using the aorta and mesenteric arteries of Sprague-Dawley rats. In mesenteric arteries contracted with phenylephrine, triclosan induced a concentration-dependent relaxation for arteries with and without endothelium. Similarly, when vasoconstriction was induced by high concentrations of potassium, a treatment with triclosan induced relaxation in the mesenteric arteries with and without endothelium. It was concluded that the mesenteric artery relaxation induced by the triclosan was independent of the endothelium. Moreover, when examining the arteries incubated with triclosan 20 min before a vasoconstriction with phenylephrine and KPSS (a high potassium physiological solution), the authors determined that triclosan significantly inhibited the contraction. In the aorta, similar results were observed as in the mesenteric arteries; however, a higher concentration of triclosan was needed for the relaxation to occur [79].

In summary, it can be stated that TCS affects the cardiovascular system in fish and rats. The effects range from vascular relaxation to an alteration of the cardiac morphology and heart rate. Furthermore, these studies also show that not all changes caused by TCS are irreversible, as presented by Cherednichenko et al., as 24 h after TCS administration, the rats recovered from some of the deficits presented. However, the molecular mechanisms underlying these cardiovascular changes remain unclear.

### 5.2. Humans

Regarding the effects of TCS in humans at the cardiovascular level, the literature is still very scarce, with only two epidemiological studies and two in vitro studies with human cells.

The first study to demonstrate this association between cardiovascular diseases and TCS was performed by Cullinan et al. [17]. In this study, the use of toothpaste with TCS (0.3% *m*/*m*) was analysed over a 5 year period in 438 patients aged up to 75 years with cardiovascular diseases (admitted to the hospital for unstable angina, abnormal electrocardiogram, and myocardial infarction). The authors observed that the prolonged use of toothpaste did not lead to an increase in adverse events or secondary cardiovascular events. Only a small decrease in time to the first serious adverse event was noted between men in the triclosan group and the placebo group [17].

Resorting to the same population sample, the same research group demonstrated the effects of using toothpaste with TCS (0.3% *m*/*m*) for 5 years on the inflammatory biomarkers of cardiovascular diseases. These biomarkers included the total cholesterol, C-reactive protein, low density lipoprotein and endothelial dysfunction. The authors observed no differences between the placebo group and the TCS group, with only slight decreases or increases in the serum levels of each of the biomarkers, except for low density lipoprotein, which decreased in the TCS group compared to the placebo group. Thus, the authors concluded that the use of toothpaste with 0.3% TCS has a minimal influence on systemic markers in patients with cardiovascular disease, showing no clinical significance in terms of reducing the risk of cardiovascular disease [80].

Regarding the in vitro studies, human embryonic stem cells (hESC) were used, as they possess the ability to differentiate into cardiomyocytes (CMs). In this study, exposure to TCS (1 µM) inhibited the differentiation of hESCs into cardiomyocytes and their spontaneous beating. This inhibition occurred through the interference of triclosan with the gene expression (of ACTC1, GATA4, and TNNT2) and with excessive methylations in the DNA [81].

A study conducted by Zhang et al., resorting to the use of HUVECs (human umbilical cord vein endothelial cells), was also performed. In the first group, the cells were treated with several doses of TCS for 24 h (0, 10, 20, 50 and 100 µM), and in the second group the cells were first treated with SC79 (PI3K/Akt activator) or MHY1485 (mTOR activator) and then treated with TCS at 50 µM for 24 h. After treatment with the activators and TCS for 24 h, the authors demonstrated that these activators were not affected by the dysfunction caused in HUVECs. In order to verify if TCS induced apoptosis in the HUVECs, a LDH test was performed demonstrating an increase in the membrane permeability, and in the caspases 3/7 activity. The levels of BAX and Bcl-2 were also calculated, where the Bcl-2 and BAX levels decreased and increased, respectively. These data demonstrate that TCS does indeed promote endothelial cell apoptosis. Then, the authors also analysed the mechanism by which TCS induced reactive oxygen species production in HUVECs and the results showed that the phosphorylation of PI3k, Akt, mTORC1 and mTORC2 was suppressed. The final test analysed the migration ability of HUVECs after exposure to TCS by wounding the culture at confluence, showing that TCS significantly reduced the regeneration ability compared to the control [82].

In short, TCS at the maximum concentrations currently allowed (e.g., 0.3% (*m*/*m*)) does not appear to have significant effects when ingested; however, in in vitro studies that have already been conducted, this compound at much higher concentrations, was found to affect the cell viability, to induce apoptosis and to decrease considerably the capacity for cell regeneration. Thus, we can conclude that studies in humans are still too scarce to infer an effect of this compound at the cardiovascular level, despite epidemiological studies pointing in that direction; therefore, it is of paramount importance to see if this compound bioaccumulates and if the doses allowed by law are really safe for human health.

## 6. Effects on the Thyroid

### 6.1. Animals

Regarding studies on thyroid function, these are still very scarce, although the first study was conducted 15 years ago.

In 2007, Crofton et al. exposed female rats (Long Evans) aged 21–23 days to 0, 10, 30, 100, 300 and 1000 mg/kg/day of TCS for 4 days to test the hypothesis that exposure to TCS altered T4 (thyroxine) levels. The authors found that the serum T4 concentration decreased by 28, 34 and 53% in the 100, 300 and 1000 mg/kg/day triclosan treatments, respectively. The study demonstrated that triclosan decreased the circulating T4 concentration in female rats [83].

In another study of triclosan toxicity was tested in two different ways. First, Wistar female rats were exposed to TCS (at 75, 150 or 300 mg/kg/day) from GD7 to PND16, and second, the offspring of unexposed females were exposed to TCS (at 0, 50 or 150 mg/kg/day) from PND 3. The T4 levels were analysed in all four groups (e.g., the control, 75, 150 and 300 mg/kg/day) at gestational day 15 (8 days after the beginning of exposure), showing a decrease by 59%, 72% and 72%, respectively, for the three concentrations of TCS, and again at postnatal day 16 (the last day of exposure), where the levels had decreased by 38%, 55% and 58%, respectively, in the females, while in the offspring no significant changes were observed. The authors concluded that TCS can decrease maternal T4 levels during gestation and lactation [11].

Resorting to male Wistar rats, Taha et al. tested the effects of compounds with an oestrogen-like structure, such as TCS, on the thyroid function. The animals were treated with two concentrations of TCS, namely, 10 and 50 mg/kg, for 60 days. The levels of the thyroid hormones, FT3 (free-triiodothyronine), FT4 (free-thyroxine), TSH (thyroid stimulating hormone), T3 (triiodothyronine) and T4, were quantified by the ELISA method. The concentration of 10 mg/kg showed no significant effect for any of the thyroid hormones; however, the concentration of 50 mg/kg led to symptoms of hypothyroidism due to changes in the T3, T4 and TSH levels [84].

Zhang et al. used Sprague-Dawley rats to better understand the mechanism by which triclosan interfered with thyroid homeostasis. After 31 days of treatment with TCS, thyroid hormones were quantified in plasma, and the results showed a decrease in all hormones inversely proportional to the concentration of the TCS administered. At the highest concentration (200 mg/kg/d), TT4 (total thyroxine), FT4, TT3 (total triiodothyronine) and FT3 were decreased by 23.1%, 20.5%, 22.0% and 19.1%, respectively. Only the TSH and TRH (thyrotropin releasing hormone) levels were unaffected. The authors also observed histological changes in the thyroid, dependent of the concentration of TCS used. Subsequently, they analysed by which pathway the TCS affected the thyroid and concluded that besides activating the JNK (c-Jun N-terminal kinase) and p38 pathways in vivo and inducing the p38 pathway from ROS in vitro, there was also an induction of the TRHr (thyrotropin releasing hormone receptor) expression through the p38 pathway, which contributed to the decrease in TPO (thyroid peroxidase). Thus, with this study it was concluded that TCS affected the thyroid homeostasis and histology through the p38/TRHr pathway mediated by TPO [85].

In a different approach, Schnitzler et al. tested whether TCS (20, 50 and 100 µg/L) affected the ontogenetic variation of thyroid hormones in developing *Cyprinodon variegatus* larvae. The total thyroid hormone concentrations were measured at 9, 12, 15 and 18 days post-hatching (dph). The results showed that the TT4 levels varied significantly in all conditions while the control had a gradual decrease. The T4 concentrations increased up to 15 dph, which was not observed for the T3 levels or the T3:T4 ratio. The peak of TT3 observed at the 20 µg/L of TCS concentration occurred 2 days after the control, being indicative of the metamorphosis climax in *Cyprinodon variegatus* [86].

In summary, TCS decreases the serum levels of T4, T3 and TSH in rats, affecting the thyroid homeostasis and histology and leading to symptoms of hypothyroidism. The same was not found in larvae where the T4 levels increased and there were no significant changes in the other hormones; thus, further studies are needed in this area as the effects of TCS on the thyroid function in animals are not yet fully defined.

### 6.2. Humans

As for the TCS effects on human thyroid function, the few studies performed so far seem to show no adverse effects.

In 2012, the use of toothpastes containing triclosan (0.3% *m*/*m*) for 4 years was analysed and the thyroid effects were observed. All 132 patients, aged up to 75 years old, had a history of hospital admission for myocardial infarction, unstable angina, and abnormal electrocardiogram. The authors measured the TSH, FT4, FT3, anti-TGb and anti-thyroid peroxidase antibody levels; however, no significant association was observed [87].

From the Health Outcomes and Measures of the Environment (HOME) study 2003–2006, Braun et al. analysed the relationship between maternal (202 women), neonatal (274 infants) and childhood (153 children) urinary TCS concentration and the corresponding serum thyroid hormone levels. The concentration of TCS in urine at 16 weeks of gestation decreased slightly until birth; however, the opposite occurred in children aged between 1–3 years. At 16 weeks’ gestation, there was no significant association between the TCS levels and thyroid hormone concentration. At the time of delivery, there was an inverse relationship between TCS and the TT4 and FT3 levels; however, due to the size of the population that was used during the study, the authors could not state that an exposure to TCS would affect the TT4 and FT3 levels [88].

Koeppe et al., using NHANES (National Health and Nutrition Examination Survey) data from 2007–2008, counted the serum levels of both free and total T3 and T4, thyroglobulin and TSH in 352 adolescents (aged 12–19 years) and 1479 adults (aged 20 years and older), and compared them with TCS concentrations measured in the urine samples collected. A positive and significant relationship between TCS and the T3 concentrations was observed only in the adolescents [89].

Berger et al. analysed the relationship between the presence of different chemicals, including TCS, with the serum levels of thyroid hormones in 338 pregnant women (aged 18–45 years) and 364 new-borns. The levels of TT4, FT4 and TSH were measured in the pregnant women while in the new-borns, only the TSH was counted. The results showed an inverse relationship between the urinary TCS concentration and the serum TT4 levels; however, no relationship was found between the urinary TCS concentrations and the TSH levels in the new-borns. It was, therefore, concluded that exposure to TCS may influence thyroid hormone levels during pregnancy [90].

More recently in 2019, the TSH, FT4, TT4, FT3, TT3, thyroid peroxidase antibody (TPOAb) and thyroglobulin antibody (TgAb) levels were measured in pregnant women at week 10 of gestation. There was no association between triclosan with the absolute TSH, FT4 or FT3 concentrations. There was no significant association between TCS and thyroid function throughout pregnancy. The association of TCS with the TSH or FT4 also had no changes with a variation of TPOAb. The association of FT4 with the TSH did not change with the various concentrations of TCS [91]. Thus, the authors concluded that TCS did not affect the thyroid function.

In a study conducted by the Korean National Environmental Health Survey (KoNEHS), the relationship between TCS exposure and thyroid hormones (namely, TT3, TT4 and TSH) was tested. The study population consisted of 2630 men and 3360 women, who provided blood and urine samples. There was a positive association between TCS exposure and the serum TSH levels in the women; however, in the men, no significant changes were observed. As for the serum levels of TT3 and TT4, no changes were noted in either the women or men. These results lead to postulating that TCS may interfere with thyroid function; however, more studies are needed to complement the limitations of this study [92].

In a similar study, using data from the environment and reproductive (EARTH) study, Skarha et al. analysed the interaction between urinary TCS levels with the serum levels of thyroid function biomarkers (namely, TSH, T4, T3, TPOAb and TgAb) in 317 women. They reached the same conclusions as Koeppe et al., with the observed urinary TCS concentrations having had no significant association with the biomarkers of the thyroid function [93].

In summary, most of the studies reviewed did not observe any changes in the thyroid function upon exposure to TCS, with only one study showing an inverse association between the TCS concentration and T4 levels; thus, considering that the lack of associations may be related to a low daily exposure to TCS, more studies are needed to determine the effects of TCS, since the studies performed so far are contradictory.

## 7. Conclusions and Future Perspectives

From all the studies presented in this review, we can conclude that in animals, TCS has clear effects; however, the same is not verified in humans, which may be due to a greater complexity, a lower TCS exposure or simply due to the difficulty in conducting in vivo studies.

Regarding the effects on the reproductive system, TCS similarly affects both animals and humans. In animals, TCS causes morphological changes in the male rat sexual organs, an increased fertilization time in fish and gestation time in rats and causes spontaneous abortions in sheep by altering the oestrogen sulphotransferase activity. While in humans, of the most common effects, alterations of the sex hormone levels stand out, and also the incidence of spontaneous abortion due to the alteration of oestrogen sulphotransferase levels as described in animals. It is also possible to observe that TCS affects both the ovarian reserve and sperm morphology and motility.

In the cardiovascular studies, as far as animals were concerned, TCS caused cardiac deficits in fish and mice; however, these were reversible in mice after 24 h. It also induced the relaxation of rat arteries and a long exposure to TCS inhibited their contraction. In humans, more specific conclusions were only drawn in vitro, in which TCS inhibited the differentiation of hESCs and had the ability to induce apoptosis in HUVECs.

Finally, from the studies analysing the thyroid function, various concentrations of TCS were tested in animals, leading to the conclusion that TCS decreased the circulating levels of T4, T3 and TSH and altered the morphology of the thyroid gland; however, in humans, the effects were inconclusive, since the studies analysed had a very small population sample with contradictory results, which reveals the need for further studies relating the TCS effects for the thyroid gland.

Thus, we can conclude that triclosan continues to be poorly studied, especially at the cardiovascular and thyroid levels. In this sense, it is essential to carry out more studies in vitro, ex vivo and in vivo on the effects of TCS on reproduction, especially on male and female fertility, on cardiovascular effects and on the relationship of thyroid hormone levels with physiological concentrations of TCS. Even at a low percentage, humans are still exposed to this endocrine disruptor on a daily basis; therefore, more epidemiological studies with larger population samples, and in different age groups and different countries are needed to really understand what effects TCS has on human health.

In summary, we will only know the true danger, or lack thereof, after this endocrine disruptor is further studied, especially in humans, as it is still a highly used chemical in cosmetic products and disinfectants in the European Union.

## Figures and Tables

**Table 1:** Triclosan general properties [26,27,28,29].

**CAS No.**	3380-34-5
**Chemical structure**	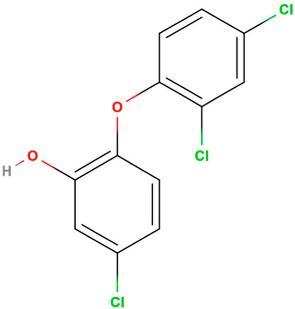
**Molecular formula**	C_12_H_7_Cl_3_O_2_
**Commercial name**	Irgasan DP 300, FAT 80′023, CH 3565, GP41-353, Irgacare MP, Lexol 300, Cloxifenolum e Ster-Zac
**Form**	Powder or crystalline powder
**Colour**	White
**Applications**	Antimicrobial, antiseptic, and preservative
**Nature**	Hydrophobic/lipophilic
**Molecular weight**	289.5 g/mol
**Density**	1.49 g/cm^3^
**Dissociation constant (pKa) (20 °C)**	7.9
**Henry constant (Hc) (atm mol^−1^** **·** **m^−3^)**	1.5 × 10^−7^ (25 °C)
**Octanol-water partition coefficient (log K_ow_)**	4.8
**Sorption coefficient (K_oc_)**	18,408
**Vapour pressure**	4 × 10^−6^ Pa (mm Hg a 20 °C)
**Triclosan degradation products**	Methyl-TCS, dioxins, chlorophenol, chloroform

**Table 2:** Solubility of triclosan in different solvents [27,28].

Solvent	Solubilities at 25 °C (g Triclosan/100 g Solvent)
Distilled water (20 °C)	0.001
Acetone	>100
Ethanol 70% or 95%	>100
Isopropanol	>100
Propylene glycol	>100
Hexane	8.5
Tween 20	>100
Glycerine	0.15

**Table 3:** Concentration of triclosan in personal care products.

Type of Product and Category	Triclosan Concentration (%)	References
**Oral hygiene**		
Toothpaste	0.3	[6]
Mouthwash	0.03	[36]
**Skin washing products**		
Liquid soap	0.1 to 0.45	[37]
Shower gel	0.3	[28]
Dishwasher	0.1	[3]
**Products applied to the skin**		
Body lotion	0.3	[28]
Facial moisturizer	0.3	
Deodorant	0.3	

**Table 4:** TCS concentrations in biological fluids.

Fluid	Concentration (nM)	Country	Reference
Serum	4.1–41.4	Spain	[52]
Plasma	0.0035–1200	Australia, Sweden	[35,53]
Urine	8.3–13090	USA	[53,54]
	0.56 ± 1.8 (non-obese)	India	[55]
	0.16 ± 0.27 (obese)		
	1.1–7.3	Spain	[52]
	0.51 ± 0.53	USA	[56]
Breast milk	0.86–7.3	Spain	[52]
	0.062–252	USA, Australia, Sweden	[33,53]

## Data Availability

Not applicable.
